# BMI, Alcohol Consumption and Gut Microbiome Species Richness Are Related to Structural and Functional Neurological Abnormalities

**DOI:** 10.3390/nu13113743

**Published:** 2021-10-23

**Authors:** Corinna Geisler, Jil Pankoke, Kristina Schlicht, Carina Knappe, Nathalie Rohmann, Katharina Hartmann, Ute Settgast, Kathrin Türk, Anna Katharina Seoudy, Andre Franke, Stefan Schreiber, Dominik M. Schulte, Matthias Laudes

**Affiliations:** 1 Institute of Diabetes and Clinical Metabolic Research, University Hospital Schleswig-Holstein, 24105 Kiel, Germany; corinna.geisler@uksh.de (C.G.); jilpankoke@web.de (J.P.); kristina.schlicht@uksh.de (K.S.); carina.knappe@uksh.de (C.K.); nathalie.rohmann@uksh.de (N.R.); katharina.hartmann@uksh.de (K.H.); ute.settgast@uksh.de (U.S.); kathrin.tuerk@uksh.de (K.T.); AnnaKatharina.Seoudy@uksh.de (A.K.S.); dominik.schulte@uksh.de (D.M.S.); 2 Division of Endocrinology, Diabetes and Clinical Nutrition, Department of Internal Medicine 1, University Hospital Schleswig-Holstein, Campus Kiel, 24105 Kiel, Germany; stefan.schreiber@uksh.de; 3 Institute of Clinical Molecular Biology, University Hospital Schleswig-Holstein and University of Kiel, 24105 Kiel, Germany; a.franke@mucosa.de

**Keywords:** gut–brain axis, neurological health, gut microbiome, nutrition

## Abstract

**Background:** The incidence of neurological diseases is increasing throughout the world. The aim of the present study was to identify nutrition and microbiome factors related to structural and functional neurological abnormalities to optimize future preventive strategies. **Methods:** Two hundred thirty-eight patients suffering from (1) structural (neurodegeneration) or (2) functional (epilepsy) neurological abnormalities or (3) chronic pain (migraine) and 612 healthy control subjects were analyzed by validated 12-month food frequency questionnaire (FFQ) and 16S rRNA microbiome sequencing (from stool samples). A binomial logistic regression model was applied for risk calculation and functional pathway analysis to show which functional pathway could discriminate cases and healthy controls. **Results:** Detailed analysis of more than 60 macro- and micronutrients revealed no distinct significant difference between cases and controls, whereas BMI, insulin resistance and metabolic inflammation in addition to alcohol consumption were major drivers of an overall neurological disease risk. The gut microbiome analysis showed decreased alpha diversity (Shannon index: *p* = 9.1× 10^−7^) and species richness (*p* = 1.2 × 10^−8^) in the case group as well as significant differences in beta diversity between cases and controls (Bray–Curtis: *p* = 9.99 × 10^−4^; Jaccard: *p* = 9.99 × 10^−4^). The Shannon index showed a beneficial effect (OR = 0.59 (95%-CI (0.40, 0.87); *p* = 8 × 10^−3^). Cases were clearly discriminated from healthy controls by environmental information processing, signal transduction, two component system and membrane transport as significantly different functional pathways. **Conclusions:** In conclusion, our data indicate that an overall healthy lifestyle, in contrast to supplementation of single micro- or macronutrients, is most likely to reduce overall neurological abnormality risk and that the gut microbiome is an interesting target to develop novel preventive strategies.

## 1. Introduction

Neurological diseases are a leading cause of disability and death worldwide. The burden of neurological diseases has increased over the past 25 years as a result of demographic change [1]. Researchers assumed that the number of patients with neurological diseases will increase steadily in the coming years, underlining the importance of examining lifestyle-associated factors for future development of targeted preventive strategies [2].

Modifiable lifestyle factors that cause a neurological disease are very diverse and not completely investigated. In particular, the influence of nutrition on the development and progression of neurological diseases is a focus of current research [3]. In addition, the intestinal microbiome as an additional modifier of nutritional factors has received scientific attention in neuroscience as a relevant system that transmits nutritional signals into the human physiology in terms of a “nutrition-microbiome-host metabolic axis”. Indeed, especially for neurodegenerative diseases, e.g., Parkinson’s disease (PD), researchers currently assume that the pathology starts in the gut by altering the intestinal autonomous nervous system [4,5].

In healthy subjects, the gut microbiome contributes to important physiological processes, such as protection against pathogens, activation of the immune system, digestion of food components and production of vitamins [6]. Furthermore, microbes have the ability to produce different metabolites, such as short-chain fatty acids, neurotransmitters, immune mediators and hormones [7]. These metabolites reflect a bidirectional communication network, known as the “gut–brain axis”. The diversity of microbes and their metabolites are thus suspected to influence gut motility and permeability, immune function [8,9,10], brain neurochemistry and emotional behaviors [11,12]. Several studies deal with the influence of microbes in the development and progression of neurological diseases, as well as the mechanisms involved in the communication between the gut–brain axis.

Preventive effects of dietary components for neuroprotection are also in the current focus of research. It is suspected that omega-3 fatty acids and vitamin D control the synthesis of serotonin and influence the development and symptom relief of neuropsychiatric disorders [13]. Dietary vitamin B3 (niacin) has been associated with both gut microbiome abnormalities and neuronal dysfunction of the central nervous system. In patients with PD, the precursor nicotinamide riboside of coenzyme NAD (nicotinamide adenine dinucleotide) may be important for mitochondrial maintenance [14].

In the present study, we examined *n* = 612 healthy controls and *n* = 238 human subjects suffering from different but common neurological disease groups: (1) structural neurological abnormalities (SNA; neurodegeneration), (2) functional neurological abnormalities (FNA; e.g., epilepsy) and (3) chronic pain (CP; e.g., migraine). The aim of the present study was to identify nutritional and microbiome factors related to structural and functional neurological abnormalities to optimize future preventive strategies. The reason not to focus on a specific neuro-disease was due to our aim to identify common nutrition and/or microbiome factors to preserve neurological health in general, which might be targeted in the future in preventive educational programs.

## 2. Materials and Methods

### 2.1. Study Cohorts and Study Design

For the present investigation, a subpopulation was generated, based on the Food Chain Plus (FoCus) cohort, which has been previously reported [15,16]. Subjects were recruited at the University Hospital Schleswig Holstein (UKSH), Campus Kiel (Germany). Within this project, a total of 2000 subjects were recruited from 2011 to 2015 and combined to form the cohort. The subjects were randomly recruited by the regional registration office and further via the obesity outpatient clinic of the Clinic for Internal Medicine I of the UKSH in Kiel. Five hundred subjects were classified as obese with a BMI over 30 kg/m^2^. The subjects underwent a medical phenotyping program that used a medical questionnaire, anthropometric measurements and analysis of markers in blood samples. Furthermore, stool samples for analysis were available, and a food frequency questionnaire (FFQ) was used once to obtain information on nutritional behavior over a time period of 12 months. The FFQ was used twice in a subsample of 200 study subjects to evaluate the validity of the FFQ. Informed consent was obtained from all subjects involved in the study. Before its commencement, the study was approved by the local ethics committee of the Department of Medicine of Kiel University (A156-03, 28 July 2011) and was registered under the clinical trial number DRKS00005285 (Food Chain Plus (FoCus) cohort) at the German Clinical Trials Register in Cologne.

The first 5-year follow-up was finished in 2020; 819 (57.5%) out of 1424 subjects completed the first follow-up, 514 subjects declined to re-participate, 44 subjects died and 47 subjects were addressed unknown. In total, complete data sets of 735 subjects were available after the first follow-up period. These data are not included in the present analysis.

### 2.2. Anthropometric Characteristics

Anthropometric data were assessed during clinical examination by the clinical staff. Weight was determined to the nearest 0.1 kg using a body composition monitor (Type BC-418 MA, Tanita Corporation, Tokyo, Japan).

### 2.3. Medical Questionnaire

A medical questionnaire (version 1. 1 dated 18 July 2011) was presented to the subjects. As a result, a case–control study population was formed with a total of 850 subjects (575 women and 275 men) including 238 cases with neurological diseases (184 women and 54 men) and 612 healthy controls (391 women and 221 men). The group of neurological cases was divided into four subgroups: (1) structural neurological abnormalities (SNA; neurodegeneration), (2) functional neurological abnormalities (FNA; epilepsy), (3) chronic pain (CP; migraine) and (4) others (these probands gave information on the presence of neurological symptoms for which the diagnostic work-up was not completed at the time of answering the medical questionnaire). Specific neurological diseases were ascertained during the medical questionnaire and additionally checked by typical medication intake if the participant reported “I have another neurological disease”.

### 2.4. Dietary Assessment

The recording of dietary behavior and nutrient intake was carried out by a self-completed, semi-quantitative dietary frequency questionnaire, according to the European Prospective Investigation into Cancer and Nutrition (EPIC)-Potsdam protocol, and analyzed by EPIC-soft as reported earlier [17,18]. The single nutrient intakes of the subcohort were compared with the D-A-CH “Reference values for nutritional intake” used in Germany [19]. Main macronutrients (e.g., carbohydrates, fat and protein) were calculated as energy percent (according to their energy density). Energy-adjusted intakes of all other nutrients were calculated by adding the mean nutrient intake to the residual derived from the regression analysis [20]. The data are presented in absolute values or percentages of energy (E%).

### 2.5. Biochemical Analyses

Blood samples were obtained by venipuncture after an overnight fast for biochemical analysis of metabolic and inflammatory markers. All markers were analyzed in the central laboratory of the university hospital in Kiel: C-reactive protein (CRP) and lipoprotein-a by immunoturbidimetry (Roche/Hitachi cobas c systems; Roche Diagnostics GmbH, Mannheim, Germany), fasting glucose by glucose-hexokinase-ultraviolet test (Roche/Hitachi cobas c systems, Roche Diagnostics International GmbH, Mannheim, Germany), fasting insulin (ECLIA; Elecsys system; Roche Diagnostics International GmbH, Mannheim, Germany) as well as interleukin-6 (IL-6) by electrochemiluminescence immunoassay (Elecsys^®^ IL-6, cobas systems; Roche Diagnostics International GmbH, Mannheim, Germany) and triglycerides by enzymatic test (Roche/Hitachi cobas c systems, Roche Diagnostics International GmbH, Mannheim, Germany). Homeostatic model assessment for insulin resistance (HOMA-IR) was calculated (fasting glucose (mg/dL) × fasting insulin (μU/mL)/405) to determine insulin sensitivity.

### 2.6. Analysis of the Intestinal Microbiome

Currently, the definition of the term microbiome is not actually standardized through different study designs. For our present work, we decided to use the term microbiome as described and defined by Berg et al. [21]. Therefore, in our manuscript, microbiome is defined as the characteristic microbial community of the gut.

For the purpose of gut microbiome analysis, stool samples were collected and stored at −80 °C until further processing. Laboratory work and quality control as well as normalization and taxonomic classification were performed by the Institute of Clinical Molecular Biology (IKMB), Kiel. As described in detail by Heinsen et al. [22], the microbiome analysis was divided into sample preparation/extraction, sequencing, amplification, quality control and bioinformatic analysis.

### 2.7. DNA Extraction, 16S rDNA Sequencing and Quality Control

In order to identify bacteria within the proband gut microbiomes, DNA sequencing of the variable regions V1/V2 of bacterial 16S rRNA genes was performed as described by Kozich et al. [23]. Here, bacterial DNA was automatically extracted using the QIAamp DNA stool mini kit and the QIAcube technology from Qiagen (Hilden, Germany). After defrosting, 200 mg from each stool sample were transferred to 0.70 mm Garnet Bead tubes from Dianova (Hamburg, Germany), then the tubes were each filled with 1.1 mL of stool lysis buffer (ASL). The SpeedMill PLUS technology (Analytik Jena, Jena, Germany) was used for bead beating at 50 Hz for 45 s, and finally samples were heated to 95 °C for 5 min and prepared for sequencing following the manufacturer’s protocol. The variable regions of interest were amplified in a dual-barcoding approach [24] using the primers 27F and 338R in a polymerase chain reaction (PCR); the related products were normalized with the SequalPrep Normalization Plate Kit (Thermo Fischer Scientific, Waltham, MA, USA) and pooled equimolarly. Then, 16S rRNA gene sequencing was conducted using the Illumina MiSeq technology (Illumina Inc., San Diego, CA, USA).

For controlling the quality of the sequencing products, further steps were performed. Demultiplexing based on 0 mismatches in the barcode sequences was carried out by allowing no mismatches; forward and reverse reads were merged using the FLASH software [25] with an allowed overlap of reads of 250 to 300 bp. Low-quality sequences were filtered out by excluding sequences with >5% nucleotides with quality score <30. Chimeras were identified with the program UCHIME [26] and were excluded from the data set.

### 2.8. Normalization and Taxonomic Classification

Sequences were clustered into operational taxonomic units (OTUs) with a sequence identity threshold of 97% using the program UPARSE [27], representing species level. The SINTAX CLASSIFIER [28] was used to carry out the taxonomic assignment of OTUs. In total, six different taxonomic levels were analyzed: domain, phylum, class, order, family and genus. For each sample, 10,000 sequences were randomly selected to form a taxon-by-sample abundance table.

### 2.9. Statistical Analysis of the Microbiome Data

Statistical calculations were performed using the program R (version 4.0.2 and 4.0.3; R Core Team (2021). R: A language and environment for statistical computing. R Foundation for Statistical Computing, Vienna, Austria. URL https://www.R-project.org/.), RStudio (version 1.3.1093; RStudio Team (2020). RStudio: Integrated Development Environment for R. RStudio, PBC, Boston, MA, USA. URL http://www.rstudio.com/.) and the R packages “Microbiome” (version 1.12.0; Leo Lahti et al., microbiome R package. URL: http://microbiome.github.io) and “pyloseq” (version 1.34.0; phyloseq: An R package for reproducible interactive analysis and graphics of microbiome census data. Paul J. McMurdie and Susan Holmes (2013) PLoS ONE 8(4):e61217.). Species richness, alpha diversity, beta diversity and the core measurable microbiome (CMM) between cases and controls as well as subgroups were analyzed. Kruskal–Wallis test was used to identify overall subgroup differences and Wilcoxon test was used to identify differences between disease subgroups and controls (as reference group). The CMM included all microbial taxa at OTU level [29], which showed an average abundance of at least 0.5% of the total bacteria within the groups. In addition, the CMM included only bacteria that were present in more than 40% of the samples. Microbiome diversity was assessed by the use of indices for alpha and beta diversity. OTU abundances in combination without and with metric and factorial variables were used for modeling a multivariate Hurdle algorithm. Two-part Hurdle models were chosen in order to handle the excess number of zeros and overdispersion in the data by evaluating zero and non-zero abundances independently. We integrated the following potential confounding factors in all analyses with confounders (medication intake, smoking habits, alcohol, dietary fiber, docosahexaenoic acid, vitamin B12, vitamin B3, vitamin D, BMI, age and sex). A Venn diagram was created using the R package “VennDiagram” (version 1.6.20; Hanbo Chen (2018). VennDiagram: Generate High-Resolution Venn and Euler Plots. R package version 1.6.20. https://CRAN.R-project.org/package=VennDiagram) to calculate the distribution of OTUs in case and control groups. The prediction of functional profiles from 16S rRNA data was performed with the “Tax4Fun” R package (version 0.3.1) [30]. “Tax4Fun” predicts the functional capabilities of microbial communities based on 16S datasets and is applicable to outputs obtained from the QIIME application [31] against the SILVA database (version 123) [32]. The “Tax4Fun” R package was embedded in the “microeco” (version 0.4.0) [33] R package, and linear discriminant analysis (LDA) scores were computed for features differentially abundant between healthy controls and neurologic cases using the Linear discriminant analysis Effect Size (LEfSe) method [34]. LEfSe is an algorithm for high-dimensional biomarker discovery and explanation to identify genomic features e.g., pathways that can characterize the differences between biological conditions.

## 3. Statistics

Statistical analysis of the data on nutritional behavior and medical history was performed using SPSS Statistic Version 22 (SPSS Inc., Chicago, IL, USA). Missing data for variables were excluded by the “Pairwise deletion” function. The statistical significance level was set to *p* < 5 × 10^−2^. For descriptive general characteristics, data were checked for normality by using the Kolmogorov–Smirnov test and are presented as means ± standard deviations (normal distribution) or median and interquartile range (non-normal distribution). Mann–Whitney U test was used to identify between-group differences. For categorical variables, a crosstabulation was generated, and the Chi-squared test was applied using SPSS Statistic Version 22 (SPSS Inc., Chicago, Illinois, USA), R (version 4.0.2 and 4.0.3; R Core Team (2021). R: A language and environment for statistical computing. R Foundation for Statistical Computing, Vienna, Austria. URL https://www.R-project.org/.) and RStudio (version 1.3.1093; RStudio Team (2020). RStudio: Integrated Development Environment for R. RStudio, PBC, Boston, MA, USA. URL http://www.rstudio.com/.) using the “finalfit” package (version 1.0.2; Ewen Harrison, Tom Drake and Riinu Ots (2020). finalfit: Quickly Create Elegant Regression Results Tables and Plots when Modelling. R package version 1.0.2. https://CRAN.R-project.org/package=finalfit). The R package “Hmisc” (version 4.50; Frank E Harrell Jr, with contributions from Charles Dupont and many others. (2021). Hmisc: Harrell Miscellaneous. R package version 4.5-0. https://CRAN.R-project.org/package=Hmisc) was used to generate correlation matrices. There are numerous factors that influence the composition of the intestinal microbiome and the gut–brain axis [7,13,14,35,36]. Thus, we integrated the following potential confounders in all analyses with confounders (medication intake, smoking habits, alcohol, dietary fiber, docosahexaenoic acid, vitamin B12, vitamin B3, vitamin D, BMI, age and sex). Multivariate logistic regression with analyses of odds ratio estimates were computed in R (version 4.0.2 and 4.0.3; R Core Team (2021). R: A language and environment for statistical computing. R Foundation for Statistical Computing, Vienna, Austria. URL https://www.R-project.org/.) and RStudio (version 1.3.1093; RStudio Team (2020). RStudio: Integrated Development Environment for R. RStudio, PBC, Boston, MA, USA. URL http://www.rstudio.com/.) using the “finalfit” package (version 1.0.2; Ewen Harrison, Tom Drake and Riinu Ots (2020). finalfit: Quickly Create Elegant Regression Results Tables and Plots when Modelling. R package version 1.0.2. https://CRAN.R-project.org/package=finalfit). For logistic regression analyses, some of the used variables were categorized: BMI subgroups were defined by general WHO standards [37], sex (men/women), medication intake (no/yes) and smoking habits (never, <3 month, former and current). Alcohol consumption was calculated as alcohol units per day (1 unit equal to 10 g alcohol), and all further phenotype and nutrition data were continuous variables.

## 4. Results

### 4.1. Characterization of Clinical Biochemistry and Anthropometric Data

The study population included 238 cases with different neurological diseases and 612 healthy controls (for details, please see Table 1).

In total, data of 575 (67.6%) women and 275 (32.4%) men were used for analysis. The overall mean age was 47.23 ± 14.34 years, and the groups were not significantly different in age. The mean BMI was significantly higher in the cases than in the controls (*p* < 1 × 10^−3^). According to the guideline values for evaluating body weight in relation to height (BMI < 25 kg/m^2^), the mean BMI values of the cases and controls were above the limit of abdominal normal weight [37]. One third of the cases were morbidly obese (Table 1), whereas approx. 45.0% of the controls showed a normal weight. More controls were overweight when compared to cases, but cases were more obese (Table 1). Among cases, BMI was evenly distributed between different neurological abnormalities (data not shown). Furthermore, cases were characterized by significantly higher levels of glucose (*p* < 1 × 10^−3^), insulin (*p* < 1 × 10^−3^), HOMA-IR index (*p* < 1 × 10^−3^), triglycerides (*p* < × 10^−3^), CRP (*p* < 1 × 10^−3^), IL-6 (*p* < 1 × 10^−3^) and lipoprotein-a levels (*p* < 1 × 10^−3^) compared to the controls (Table 1). Neurological cases showed a higher prevalence (19.1%; *p* < × 10^−3^) of diabetes (all types), high blood lipids (36.4%; *p* < 1 × 10^−3^), hypertension (55.3%; *p* < 1 × 10^−3^), inflammatory bowel disease (6.8%; *p* < 1 × 10^−3^) and inflammatory bowel syndrome (7.7%; *p* < 1 × 10^−3^).

### 4.2. Characterization of Micro- and Macronutrients

According to official recommendations, the protein intake should be 10–15% of the total energy requirement (E%) [19]. In our cohort, total protein intake of all subjects was within the range of the reference values (Table 2). There were no significant differences between groups in total protein (Table 2) and amino acid intake (Table S1). The total study population had a higher median total fat intake (Table 2) than recommended (reference value for total fat intake is 30 E% [19]). The intake of saturated and polyunsaturated fatty acids (ω-6 and ω-3) was also above the reference values (7 E%, 2.5 E% and 0.5 E% [19,38]) in cases and controls. In addition, there were significant differences between groups in intakes of polyunsaturated fatty acids (*p* < 1 × 10^−2^) and octadecadienoic acid/linoleic acid (*p* < 1 × 10^−2^) (Table 2). The results show that all subjects had an inadequate total carbohydrate intake below the recommendation of 55 E% [19] (Table 2). Furthermore, there was a significant difference between cases and controls regarding total carbohydrate intake (*p* < 2 × 10^−2^) (Table 2). Dietary fiber intake was below the recommendation of 30 g/day in cases and controls [19] (Table 2). Both groups covered only 69% of the daily recommended dietary fiber intake, but cases and controls were not significantly different (Table 2). Alcohol intake of the entire study population was 2.2 E%, with higher alcohol consumption in controls when compared to cases (*p* < 1 × 10^−3^) (Table 2), but both groups were below the guideline values considered safe for health [19]. Salt intake was below the reference value of 6 g/day (Table 2) and not significantly different between cases and controls. The micronutrient intake showed that the median calcium intake was inadequate and below the reference value of 1000 mg/day (Table 2) [19]. This was also true for vitamin B9 (total folic acid) and vitamin B5 (pantothenic acid) [19]. Only vitamin D intake was significantly different between cases and controls (*p* < 5 × 10^−2^) (Table 2), but intake was within the recommendation (3–4 μg/day) of dietary vitamin D intake [19].

## 5. Neurological Cases and Microbiome

In total, data on the microbiome analysis of 688 subjects were available. These were 223 cases and 465 controls. Disease subgroups were also used for microbiome analyses.

### 5.1. Core Measurable Microbiome (CMM)

A CMM of 186 operational taxonomic units (OTUs) was identified for controls and 149 OTUs for cases. Merging both groups resulted in 191 OTUs. The merged OTUs for the subgroups accounted for 375 OTUs in total. The CMM was assignable to five phyla: *Actinobacteria*, *Bacteroidetes*, *Firmicutes*, *Proteobacteria*, and *Verrucomicrobia*.

A Venn diagram (Figure S1) showed that there was an overlap of 144 OTUs of differential abundance in cases and controls. Cases presented 5 OTUs (mainly *Proteobacteria*) that were solely observed in this group, whereas 42 OTUs were solely observed in healthy controls (mainly *Firmicutes*).

We used hurdle models for further analysis of CMM differences between cases and controls and for the identification of potential marker species. A basic model without potential confounding variables showed that 51 OTUs were significantly (*p* < 5 × 10^−2^) different between the cases and controls. The main phylum was *Firmicutes* (41 OTUs) and the minor amounts were *Bacteroidetes* (6 OTUs) and *Proteobacteria* (4 OTUs). Cases showed a decreased (*p* < 5 × 10^−2^) count in all OTUs except three OTUs of *Proteobacteria* (family: *Enterobacteriaceae*). After adjustment for the number of OTUs (FDR correction) and potential confounders (medication intake, smoking habits, alcohol, dietary fiber, docosahexaenoic acid, vitamin B12, vitamin B3, vitamin D, BMI, age and sex), there were no longer significant differences observed between cases and controls in OTUs, either in the count or in the binomial part of the hurdle model.

Hurdle models were also analyzed in the subgroups. Models both without and with potential confounders showed no significant differences between subgroups in OTUs, either in the count or in the binomial part of the hurdle models.

### 5.2. Differences in Alpha Diversity between Neurological Cases and Healthy Controls

**Figure 1:**
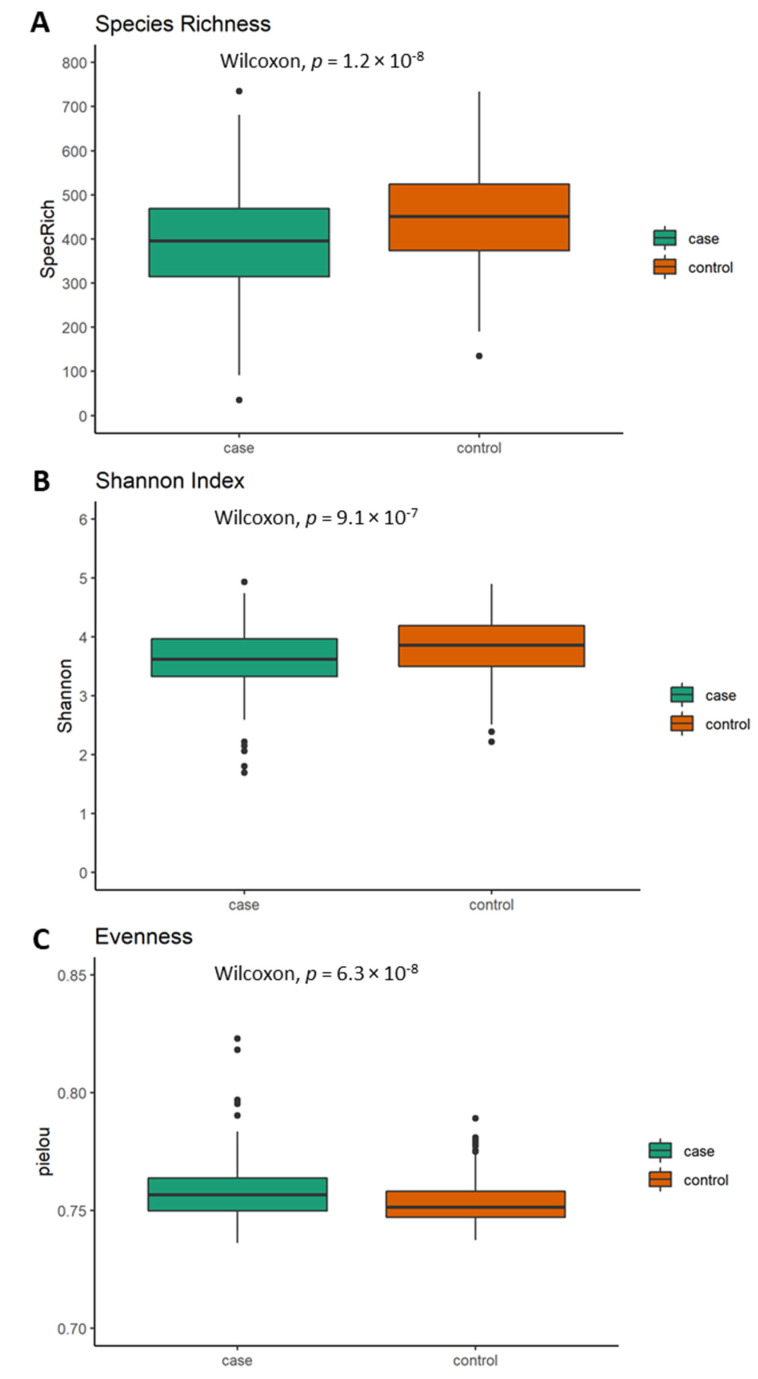
Alpha diversity and evenness differences between cases and controls displayed through three indices: (**A**) species richness, (**B**) Shannon index and (**C**) evenness (Pilou); (spot = outlier, Wilcoxon test; *p* < 5 × 10^−2^).

With respect to the alpha diversity measures, cases presented significantly lower species richness (*p* = 1.2 × 10^−8^; Figure 1A), Shannon index (*p* = 9.1 × 10^−7^; Figure 1B) and higher evenness (*p* = 6.3 × 10^−8^; Figure 1C), presenting a lower variation in abundances between different taxa within the cases than controls.

**Figure 2:**
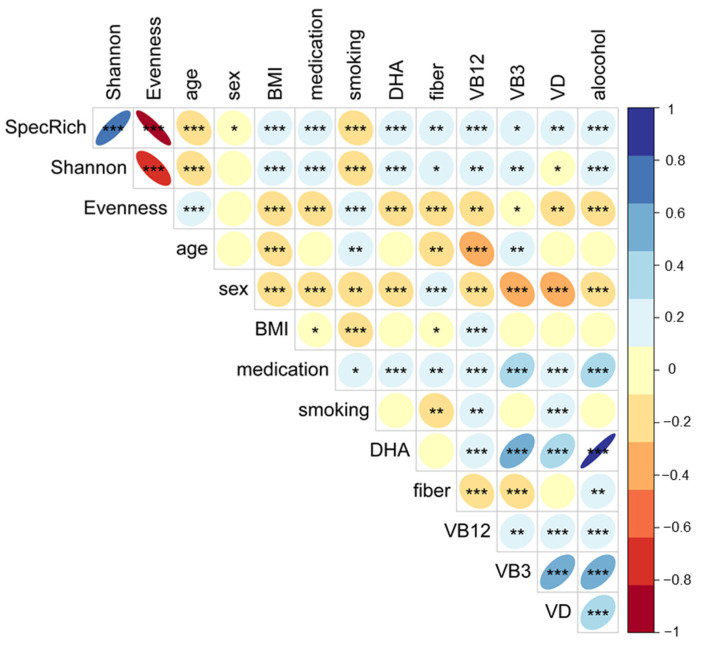
Spearman correlations between alpha diversity indices, phenotype, health and nutrition data of all subjects (* *p* < 5 × 10^−2^, ** *p* <1 × 10^−2^ and *** *p* < 1 × 10^−3^). Continuous variables: species richness (SpecRich), evenness, Shannon index (Shannon), age (in years), BMI (in kg/m^2^), fiber (g/day), docosahexaenoic acid (DHA, g/day), vitamin B12 (VB12, µg/day), vitamin B3 (VB3, mg/day), vitamin D (VD, µg/day) and alcohol (as percentage of energy) and categorial variables: medication (intake: no (2)/yes (1)) and smoking habits (never (1), <3month (2), former (3) and current (4)) and sex (men (1)/women (2)).

Spearman correlation analyses showed that alpha diversity indices were correlated to potential confounders (Figure 2). These confounders were subsequently included in logistic regression models to evaluate their impact on the observed significant differences in alpha diversity between cases and controls.

Species richness, Shannon index and evenness remained significantly different between cases and controls (*p* = 1.95 × 10^−4^; *p* = 1.81 × 10^−3^ and *p* = 1.47 × 10^−4^) even after using an ANOVA with potential confounders (medication intake, smoking habits, alcohol, dietary fiber, docosahexaenoic acid, vitamin B12, vitamin B3, vitamin D, BMI, age and sex).

### 5.3. Differences in Alpha Diversity between Neurological Subgroups and Healthy Controls

**Figure 3:**
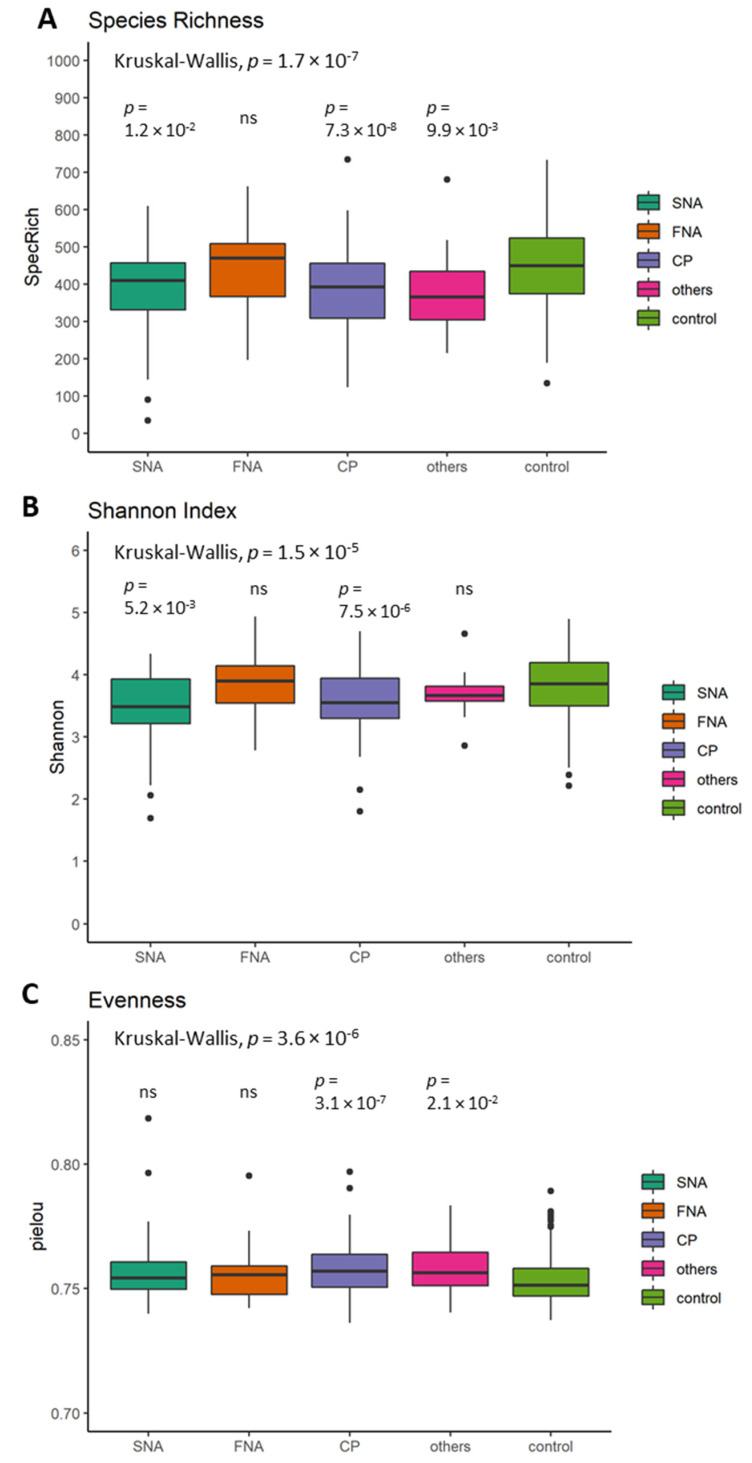
Alpha diversity and evenness differences between neurological subgroups and controls displayed through three indices (**A**) species richness, (**B**) Shannon index and (**C**) evenness (Pilou); (spot = outlier, overall and pairwise significance was tested by Kruskal–Wallis and Wilcoxon test (reference group = control); *p* < 5 × 10^−2^). SNA: structural neurological abnormalities; FNA: functional neurological abnormalities; CP: chronic pain and others (= these probands gave information on the presence of neurological symptoms for which the diagnostic work-up was not completed at the time of answering the FoCus questionnaire).

Alpha diversity in subgroups was overall significantly different in species richness (*p* = 1.7 × 10^−7^; Figure 3A), Shannon index (*p* = 1.5 × 10^−5^; Figure 3B) and evenness (*p* = 3.6 × 10^−6^; Figure 3C). When compared to the reference group (=control), CP showed significantly lower species richness (*p* = 7.3 × 10^−8^; Figure 3A), Shannon index (*p* = 7.5 × 10^−6^; Figure 3B) and a higher evenness (*p* = 3.1 × 10^−7^; Figure 3C). Species richness, Shannon index and evenness remained significantly different between subgroups (*p* = 8.59 × 10^−4^; *p* = 1.25 × 10^−3^ and *p* = 2.04 × 10^−5^) after using an ANOVA with potential confounders (medication intake, smoking habits, alcohol, dietary fiber, docosahexaenoic acid, vitamin B12, vitamin B3, vitamin D, BMI, age and sex).

### 5.4. Difference in Beta Diversity between the Neurological Cases, their Subgroups and Healthy Controls

**Figure 4:**
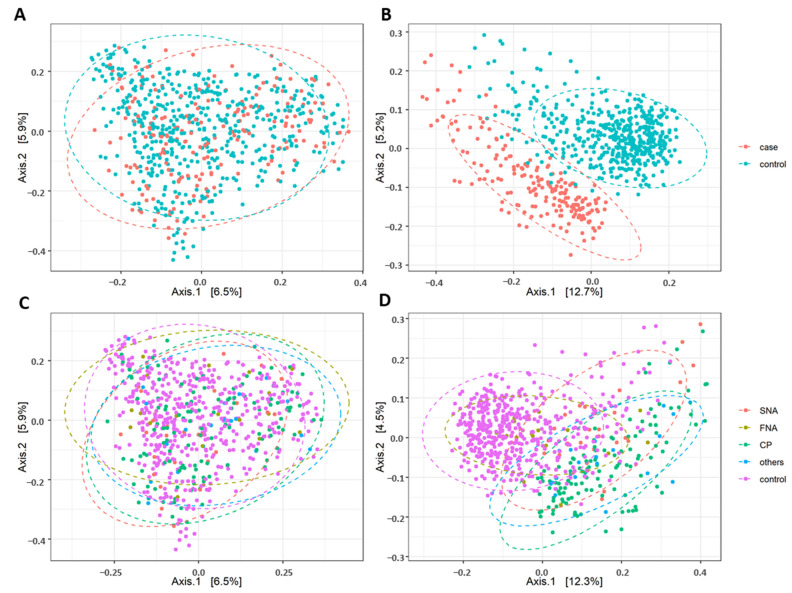
Differences between controls and cases in principal coordinate analysis (PCoA) without confounding factors in (**A**) Bray-Curtis and (**B**) Jaccard beta diversity and between neurological subgroups in (**C**) Bray–Curtis and (**D**) Jaccard beta diversity (permutation analysis of variance (PerMANOVA); all were significantly different with *p* < 5 × 10^−2^). SNA: structural neurological abnormalities; FNA: functional neurological abnormalities; CP: chronic pain and others (=these probands gave information on the presence of neurological symptoms for which the diagnostic work-up was not completed at the time of answering the FoCus questionnaire).

Figure 4 visualizes the beta diversity based on principal coordinate analysis (PCoA). Dissimilarity matrices were calculated for abundance of OTUs according to Bray–Curtis and for presence/absence of OTUs according to Jaccard. To establish significance of differences between the groups, a permutation analysis of variance (PerMANOVA) was performed. The analyses according to Bray–Curtis showed a significant difference in beta diversity between cases and controls (*p* = 9.99 × 10^−4^; r2 = 0.55%; Figure 4A) as well as for Jaccard (*p* = 9.99 × 10^−4^; r2 = 1.61%; Figure 4B). Using the neurological subgroups, Bray–Curtis and Jaccard analyses showed a significant difference in beta diversity between subgroups (*p* = 9.99 × 10^−4^; r2 = 1.05%; Figure 4C and *p* = 9.99 × 10^−4^; r2 = 2.25%; Figure 4D).

In addition, the beta diversity of the intestinal microbiome was adjusted for different confounders by constrained ordination analysis. Differences in beta diversity between cases and controls were calculated and adjusted for medication intake, smoking habits, alcohol, dietary fiber, docosahexaenoic acid, vitamin B12, vitamin B3, vitamin D, BMI, age and sex and group membership (cases/controls and respective subgroups). Bray–Curtis and Jaccard both showed significant differences in adjusted beta diversity between cases and controls as well as subgroups (see Table 3 and Table 4).

### 5.5. Prediction of Functional Profiles

**Figure 5:**
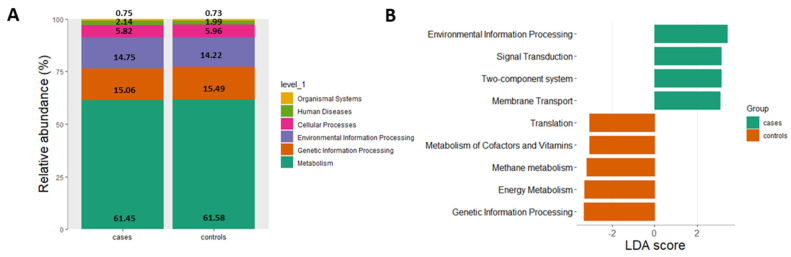
Results of the relative abundance (in %) of level 1 (**A**) functional pathways and (**B**) linear discriminant analysis (LDA) effect size (LEfSe) plot of functional pathways identified in the gut microbiomes of cases and controls. The threshold for the logarithmic discriminant analysis (LDA) score was 3 (*p* < 5 × 10^−2^). Figures and calculations were assessed by the “microeco” package (version 0.4.0) with the “Tax4Fun” package included in RStudio (version 0.3.1).

The Linear discriminant analysis Effect Size (LEfSe) algorithm, emphasizing both statistical and biological relevance, was used for biomarker discovery of functional profiles. The following results of the functional pathway analysis as assessed by LEfSe were based on 16S rRNA sequencing; thus, the results should be understood as predictive results. According to LEfSe analysis, more than 2/3 of the functional profiles were associated with metabolism in general and genetic information processing (Figure 5A). The linear discriminant analysis (LDA) effect size method was applied to compare functional profiles between controls (dark grey) and cases (light grey). The bar plot lists the significantly differential metabolic pathways based on effect size (LDA score (log 10)  of 3). Enriched profiles in controls (negative LDA score) included translation, metabolism of cofactors and vitamins, methane metabolism, energy metabolism as well as genetic information processing. The enriched profiles in cases (positive LDA score) were environmental information processing, signal transduction, two component system and membrane transport. The results of the LDA scores are presented in Figure 5B.

The LDA effect size method was also applied to compare functional profiles between subgroups. SNA was the only subgroup that showed significantly differential metabolic pathways based on effect size (LDA score (log 10) of 3), which were different from controls. Enriched profiles in SNA were lipid metabolism, infectious diseases, human diseases, carbohydrate metabolism, ABC transporter, membrane transport, two component system, signal transduction and environmental information processing (Figure S2).

### 5.6. Calculation of the Risk to Develop Neurological Diseases

**Figure 6:**
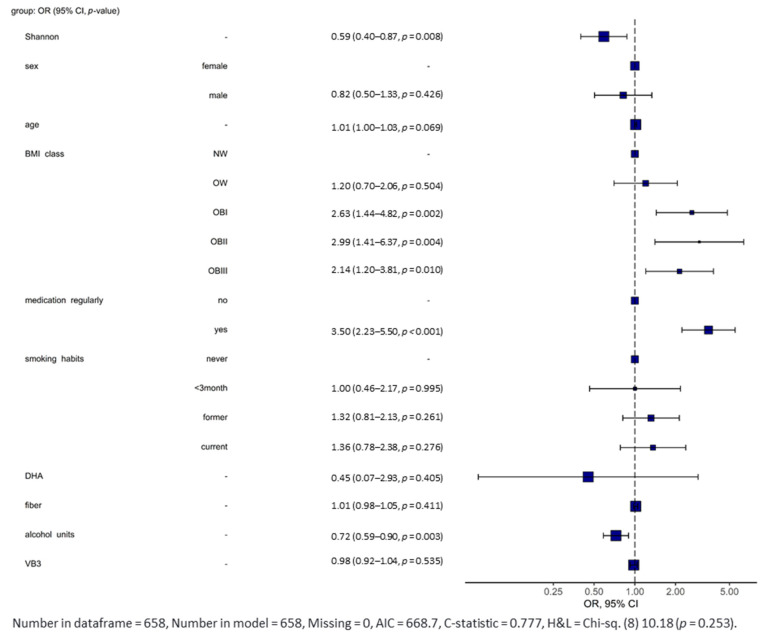
Forest plot showing the odds ratios with corresponding 95% confidence interval and *p*-values of variables used in a multivariate regression model. Twelve variables were included in the calculation, but vitamin D and vitamin B12 were excluded from this Forest plot due to their extreme odds ratios and corresponding confidence intervals. Reference category was the healthy control group. Figure and calculations were assessed by the “finalfit” package (version 1.0.2) for R in RStudio (version 1.3.1093) with *p* < 5 × 10^−^^2^. NW: normal weight (BMI 18.50 to 24.99 kg/m^2^); OW: overweight (BMI 25.00 to 29.99 kg/m^2^); OBI: obesity class I (BMI 30.00 to 34.99 kg/m^2^); OBII: obesity class II (BMI 35.00 to 39.99 kg/m^2^); OBIII: obesity class III (BMI ≥ 40.00 kg/m^2^). Reference category was at all times the first category of the corresponding categorical variable.

A binomial logistic regression was performed to determine the effect of medication intake, smoking habits, alcohol, dietary fiber, docosahexaenoic acid, vitamin B12, vitamin B3, vitamin D, BMI, age and sex and Shannon index as a marker of alpha diversity and richness to predict the likelihood of contracting neurological diseases. All variables were included in a multivariate logistic regression model, and the following variables contributed significantly as shown by Forest plot (Figure 6) in predicting neurological diseases. Vitamin D and vitamin B12 were excluded from this Forest plot due to extreme odds ratios and corresponding confidence intervals. The following variables entered the multivariate model: Shannon index (*p* = 8 × 10^−3^), obese BMI subgroups (OBI: *p* = 2 × 10^−3^; OBII: *p* = 4 × 10^−3^ and OBIII: *p* = 1 × 10^−2^), medication intake (*p* = 1 × 10^−3^) and increased units of alcohol consumption (*p* = 3 × 10^−3^). Shannon index had a beneficial effect and reduced the relative risk to develop a neurological disease by −41.0% as well as alcohol consumption (−28.0%), whereas obesity (2.14- to 2.99-fold higher risk) and medication intake (3.5-fold higher risk), as a marker for presence of non-neurological diseases, had unbeneficial effects.

## 6. Discussion

The present study aimed to identify common nutritional and microbiome signatures in structural and functional neurological abnormalities as well as chronic pain in order to develop future targeted preventive strategies. By detailed examination of macro- and micro-nutritional intake as well as 16S rRNA gut microbiome sequencing in a cohort of 238 cases with different neurological abnormalities and 612 controls, we found that (1) BMI, (2) dietary alcohol consumption and (3) gut microbiome species richness and diversity are the most relevant lifestyle modifiable factors contributing to the risk for the development of structural and functional neurological abnormalities. In addition, age was identified as the most, but not significantly important, non-modifiable factor. During the following discussion, a wide range of references will be based on results in PD and AD patients due to the fact that for migraine or epilepsy patients, the database-entered studies in these patients’ groups are very rare in comparison to PD or AD (https://pubmed.ncbi.nlm.nih.gov/ (accessed on 19 July 2021)).

Of interest in our cohort, we found that the average BMI was higher in the cases than in the healthy controls, which was unexpected because particularly in PD patients, a higher risk of developing malnutrition is reported even in early stages of the disease. On the other hand, obesity may also be promoted due to immobility and reduced total energy expenditure in neurological diseases. Patients with neurological abnormalities such as PD, epilepsy or restless leg syndrome also suffer more frequently from sleep disorders, which are known to promote weight gain and loss of fat-free mass [39]. Furthermore, in addition to BMI, our neurological cases were characterized by significantly higher levels of glucose, fasting insulin and HOMA levels, indicating insulin resistance. In that respect, it is important to mention that patients with PD [40] or epilepsy often suffer from undiagnosed insulin resistance or show a higher risk of developing type 2 diabetes [41], and that obese subjects were affected by PD twice as often as those with normal weight [40], fitting with our results.

In the present analysis, the cases with neurological abnormalities were also characterized by higher CRP, IL-6 levels and lipoprotein-a levels compared to the healthy control group. Neuropathological and neuroradiological studies suggest that a common cause of many neurological diseases is thought to be neuroinflammation of the brain [42]. Infections, trauma, and/or toxins from food can promote neuroinflammatory processes. This activates immune cells such as microglia. As a result, cytokines and chemokines such as IL-1β, IL-6 and TNF-α are released [43].

Patients with neurological diseases such as PD or migraine often suffer from abdominal discomfort. Conversely, in a case–control study, the prevalence of headache was higher in patients with inflammatory bowel disease when compared to controls (46% vs. 7%), and another study from Brazil showed that headache was the most common neurologic manifestation in IBD patients with inflammatory bowel disease [44,45]. Unger et al. reported that a reduced number of short-chain fatty acids (SCFAs) and an altered composition of the intestinal microbiome in stool samples from PD patients were detectable [46]. A randomized study showed that the Mediterranean diet had a particularly positive effect on the diversity of microbes producing SCFAs [47]. A high-fiber diet based on the Mediterranean diet can thus positively influence the number of microbes and the production of SCFAs, thereby reducing abdominal discomfort. In that respect, it should be mentioned that dietary fiber intake was below the recommendation of 30 g/day in our cohort, with no difference between cases and controls. Hence, low fiber intake, by promoting abdominal discomfort, might negatively affect the course of the disease in PD but is unlikely to be causative.

It is important to note that gut microbiota composition plays a major role in the gut–brain axis and is related to two mechanisms: indirect signaling and direct connection with the vagus nerve. Dysbiosis of the microbiome could alter the protective functions of the blood–brain barrier [48,49]. Thus, the composition of the intestinal microbiome was compared on the basis of fecal samples obtained from cases with neurological disease and healthy controls. In terms of alpha diversity, cases showed a lower Shannon index, as well as a reduced species richness, compared to controls. There were also differences between cases and controls in terms of beta diversity even when adjusted for different potential confounders, such as BMI. In a study using similar methods, independent researchers investigated to what extent the intestinal microbiome differed between PD patients and a healthy control group in northeastern China [50]. PD patients showed reduced species richness and beta diversity, as well as reduced abundance of several taxa, compared to the healthy control group [50], comparable to what we found in the present study. It has to be mentioned that another study by Li et al. [51] showed a tendency of higher Chao1 and Shannon index in the group with PD compared to the healthy control group, which is in contrast to our study. However, the study by Li et al. [51] was only related to PD and not to different neurological diseases. Because we aimed to identify common factors in nutrition and microbiome related to neurological abnormalities, we did not focus only on PD, which might explain the different findings. Mainly predictive results of functional analyses showed that cases with neurological abnormalities could be discriminated by four functional pathways from healthy controls. These pathways included, e.g., adenosine triphosphate (ATP)-binding cassette (ABC) transporters, phosphoenolpyruvate (PEP)-dependent phosphotransferase system (PTS) or different signaling pathways, e.g., nuclear factor kappa-light-chain-enhancer of activated B cells (NF-κB), wingless/integrase 1 (wnt) or vascular endothelial growth factor (VEGF) signaling. Adenosine, for example, plays a role in different functions throughout the brain, e.g., metabolism, cell signaling, neuronal signaling and inflammation [52], and it is also involved in migraine [53]. The signaling pathways are all known to be involved in neurological diseases, especially in migraine. NF-κB, for example, is a main player in regulating nerve function [54], and furthermore, the inflammatory NF-κB pathway is known to interact with wnt signaling [55], whereas VEGF is an important proinflammatory mediator, and an inhibition of signaling led to pain decrease [56]. Migraine is one of the most prevalent neurological diseases, and VEGF, for example, stimulates nitric oxide synthase and therefore increases nitric oxide levels [57]. Thus, the observed predictive functional pathways are in line with previous findings.

While in our study we were able to identify nutritional and microbiome factors as potential indicators for neurological abnormalities, our study has some limitations to consider. The EPIC 12-month FFQ used is suitable due to its high compliance and computer readability, especially for the survey of larger samples, as in our FoCus study. However, its use is also associated with limitations. For correct answering, a very good memory of the subjects is necessary. Furthermore, the response behavior is influenced by dietary habits and social trends. It cannot be excluded that under- and overreporting of food has occurred [58]. In addition, baseline data were obtained from the subjects by means of self-completed questionnaires on lifestyle and medical issues. The data are based on self-reporting by the subjects and may therefore be subject to inaccuracy.

In summary, the results of our multivariate binomial logistic regression indicate that especially obesity associated with insulin resistance and metabolic inflammation is related to structural and functional neurological abnormalities as well as chronic pain. We observed an obesity-driven higher risk to develop neurological abnormalities, e.g., a higher BMI increased the relative risk up to 2.99-fold. This is in line with findings that obesity adversely affects the central nervous system and, in particular, cognitive function. Meta-analyses have shown a strong relationship between obesity and neurological diseases [59]. There is also evidence that obesity doubles the risk of Alzheimer’s disease in comparison to normal-weight subjects, and that obesity in midlife predicts greater risk of dementia in future [35,59,60,61,62]. Alcohol consumption showed a beneficial effect in our logistic regression model. This fits the widely accepted theory that light-to-moderate alcohol intake is beneficial, while excessive drinking increases the risk of dementia [63,64]. On the other hand, one should keep in mind that a low alcohol consumption in subjects with neurological diseases could be more the consequence than the cause of neurological diseases. In our study population, 17.2% showed risky alcohol intake.

In conclusion, our data suggest that an overall healthy lifestyle might be more important in respect to developing preventive neurological strategies compared to single dietary compounds, e.g., PUFA. In addition, the observed differences in alpha and beta diversity showed that the gut microbiome might be used as a future preventive target through nondigestible food components (prebiotics) and the supplementation of bacteria (probiotics).

## Figures and Tables

**Table 1:** Characterization of the FoCus subcohort according to laboratory analytical and anthropometric factors and demographic data.

	Cases (*n* = 238)	Controls (*n* = 612)	All Subjects (*n* = 850)
Women	184 (77.3%) ^a^	391 (63.9%)	575 (67.6%)
Men	54 (22.7%) ^a^	221 (36.1%)	275 (32.4%)
Neurological diseases			
SNA	15.1%		
Parkinson’s disease	1 (0.4%)		
Tremor	7 (2.9%)		
Restless leg	26 (10.9%)		
Multiple sclerosis	2 (0.8%)		
FNA	11.3%		
Epilepsy	16 (6.7%)		
Anxiety	8 (3.4%)		
Depression	2 (0.8%)		
Sleep disorder	1 (0.4%)		
CP	62.2%		
Migraine	135 (56.7%)		
Pain	12 (5.0%)		
Neuropathy	1 (0.4%)		
Others	27 (11.3%)		
Age, year	48.49 (±13.41) ^1^	46.73 (±14.68)	47.23 (±14.34)
Weight, kg	100.10 (±33.06) *	84.82 (±26.66)	89.10 (±29.40)
Height, cm	171.18 (±8.41) *	173.57 (±8.88)	173.90 (±8.81)
BMI, kg/m^2^	34.14 (±10.85) *	28.05 (±8.22)	29.76 (±9.43)
UW	1.8% (4)	1.9% (11)	1.9% (15)
NW	22.4% (50) ^a^	44.5% (253)	38.3% (303)
OW	17.0% (38) ^a^	28.0% (159)	24.9% (197)
OBI	17.0% (38) ^a^	9.7% (55)	11.8% (93)
OBII	11.2% (25) ^a^	4.8% (27)	6.6% (52)
OBIII	30.6% (68) ^a^	11.1% (63)	16.6% (131)
Glucose, mg/dL	95.00 (88.00/105.00) *^2^	92.00 (87.00/99.00)	93 (87.00/101.00)
Insulin, µU/mL	12.20 (7.30/22.65) *	8.50 (5.80/13.10)	9.30 (6.20/15.30)
HOMA-IR	2.93(1.61/5.79) *	1.99 (1.30/3.08)	2.13 (1.37/3.60)
Triglyceride, mg/dL	119.00 (79.00/168.25) *	87.00 (66.00/125.00)	95.00 (68.75/139.00)
CRP, mg/L	2.60 (0.90/6.40) *	1.10 (0.90/2.80)	1.30 (0.90/3.70)
IL-6, pg/mL	3.65 (2.10/5.43) *	2.60 (1.50/3.80)	2.80 (1.60/4.35)
Lipoprotein-a, mg/L	104.00 (95.00/245.00) *	95.00 (93.10/200.00)	95.00 (95.00/214.25)

^a^ Statistical significance between groups and gender were tested using chi-square test, (*p* < 5 × 10^−2^); ^1^ mean (±SD); ^2^ median (25th and 75th percentiles); * statistical significance was tested using Mann–Whitney U test, (*p* < 5 × 10^−2^). SNA: structural neurological abnormalities; FNA: functional neurological abnormalities; CP: chronic pain; BMI: body mass index; UW: underweight (BMI < 18.50 kg/m^2^); NW: normal weight (BMI 18.50 to 24.99 kg/m^2^); OW: overweight (BMI 25.00 to 29.99 kg/m^2^); OBI: obesity class I (BMI 30.00 to 34.99 kg/m^2^); OBII: obesity class II (BMI 35.00 to 39.99 kg/m^2^); OBIII: obesity class III (BMI ≥ 40.00 kg/m^2^); HOMA-IR index: homeostasis model assessment of insulin resistance; CRP: C-reactive protein; IL-6: interleukin-6.

**Table 2:** Characterization of energy adjusted macro- and micronutrient intake between the groups.

	Cases (*n* = 223)	Controls (*n* = 568)	All Subjects (*n* = 791)
Protein total, E%	14.52 (13.04/15.89) ^1^	14.38 (12.96/15.79)	14.42 (12.98/15.85)
Essential amino acids, g/day	34.87 (28.48/41.37)	37.12 (33.60/41.43)	37.23 (33.65/41.29)
Arginine, g/day	4.17 (3.65/4.73)	4.09 (3.61/4.57)	4.11 (3.63/4.59)
Cysteine, g/day	1.01 (0.92/1.10)	0.99 (0.91/1.09)	1.00 (0.91/1.09)
Tyrosine, g/d	2.70 (2.41/2.93)	2.67 (2.41/2.96)	2.68 (2.41/2.95)
Methionine, g/d	1.60 (1.44/1.80)	1.61 (1.43/1.81)	1.61 (1.44/1.81)
Phenylalanine, g/d	3.18 (2.60/3.71)	3.31 (3.04/3.61)	3.33 (3.04/3.60)
Tryptophan, g/day	0.86 (0.77/0.94)	0.86 (0.78/0.94)	0.86 (0.77/0.94)
Fat total, E%	42.05 (37.74/45.25)	41.29 (37.42/44.55)	41.43 (37.60/44.82)
Saturated fatty acids, E%	36.86 (28.80/44.04)	37.74 (29.52/47.02)	37.22 (29.25/46.20)
Short-chain fatty acids, E%	0.81 (0.65/1.02)	0.86 (0.66/1.07)	0.84 (0.66/1.05)
Medium-chain fatty acids, g/d	0.76 (0.65/0.90)	0.80 (0.66/0.92)	0.79 (0.66/0.91)
Long-chain fatty acids, E%	37.57 (33.92/40.43)	36.79 (33.36/39.89)	36.90 (33.58/40.03)
Polyunsaturated fatty acids, E%	7.37 (5.93/8.47) *	7.04 (5.74/7.93)	6.86 (5.80/8.14)
Octadecadienoic acid/linoleic acid, (g/day)	14.28 (12.17/16.61) *	13.53 (11.54/15.74)	13.70 (11.70/16.02)
Octadecatrienoic acid/linolenic acid, (g/day)	1.95 (1.79/2.28)	1.93 (1.76/2.25)	1.93 (1.77/2.26)
Eicosatetraenoic acid/arachidonic acid, (g/day)	0.18 (0.15/0.22)	0.18 (0.15/0.22)	0.18 (0.15/0.22)
Docosahexaenoic acid, (g/day)	0.18 (0.13/0.26)	0.20 (0.13/0.27)	0.19 (0.13/0.26)
Monounsaturated fatty acids, E%	14.38 (13.00/16.02)	14.37 (12.86/15.75)	14.38 (12.91/15.79)
Carbohydrate total, E%	42.99 (38.98/46.98) *	41.46 (38.37/45.78)	41.79 (38.55/46.08)
Monosaccharides, g/day	49.00 (37.77/62.00)	47.83 (35.91/59.79)	48.12 (36.50/60.34)
Fructose, g/day	26.33 (20.75/34.50)	25.47 (19.75/33.59)	25.63 (20.02/33.81)
Galactose, g/day	4.23 (4.05/4.57)	4.20 (4.05/4.46)	4.21 (4.06/4.50)
Glucose, g/day	21.85 (16.69/27.43)	21.46 (15.89/26.13)	21.57 (16.25/26.56)
Dietary fiber total, g/day	21.62 (18.91/26.47)	21.38 (18.67/25.22)	21.51 (18.75/25.55)
Soluble fiber, g/day	7.14 (6.03/8.47)	6.94 (5.99/8.14)	6.96 (6.00/8.21)
Insoluble fiber, g/day	14.67 (12.69/17.61)	14.33 (12.52/16.97)	14.47 (12.55/17.22)
Alcohol, E%	1.10 (0.42/2.80) *	2.56 (1.02/5.10)	2.18 (0.72/4.58)
Table salt, g/day	5.54 (4.93/6.08)	5.46 (4.97/5.99)	5.48 (4.96/6.03)
Calcium, mg/day	865.60 (749.60/1016.90)	852.30 (737.10/989.50)	857.50 (740.50/995.90)
Vitamin B1 (thiamine), mg/day	1.76 (1.61/1.86)	1.73 (1.61/1.85)	1.74 (1.61/1.85)
Vitamin B12 (cobalamin), µg/day	5.50 (4.40/6.40)	5.70 (4.50/6.70)	5.50 (4.40/6.70)
Vitamin B2 (riboflavin), mg/day	1.51 (1.32/1.69)	1.48 (1.33/1.67)	1.49 (1.33/1.68)
Vitamin B3 (niacin), mg/day	14.21 (12.13/16.75)	14.77 (12.46/17.19)	14.64 (12.33/17.00)
Vitamin B5 (pantothenic acid), mg/day	4.55 (3.79/5.45)	4.59 (3.81/5.63)	4.58 (3.80/5.54)
Vitamin B6 (pyridoxine), mg/day	1.55 (1.40/1.75)	1.56 (1.40/1.73)	1.55 (1.40/1.74)
Vitamin B7 (biotin), µg/day	45.20 (40.30/49.80)	44.70 (39.90/50.50)	44.90 (40.00/50.50)
Vitamin B9 (free folic acid equivalent), µg/day	110.30 (94.80/130.70)	112.20 (95.30/125.70)	110.80 (95.20/126.50)
Vitamin B9 (free folic acid), µg/day	87.60 (72.10/100.00)	84.60 (71.10/96.10)	84.30 (71.70/96.80)
Vitamin B9 (total folic acid), µg/day	275.00 (248.90/312.40)	276.20 (249.90/305.50)	276.00 (249.80/308.30)
Vitamin C (ascorbic acid), mg/day	123.37 (98.28/162.98)	118.09 (95.73/157.92)	119.25 (96.36/158.86)
Vitamin D (calciferols), µg/day	3.70 (2.70/4.90) *	4.40 (2.90/5.20)	3.90 (2.80/5.10)
Vitamin E (tocopherol equivalent), mg/day	14.08 (11.61/15.80)	13.69 (12.28/15.23)	13.80 (12.36/15.39)
Vitamin K (phylloquinone), µg/day	314.50 (274.90/357.00)	305.30 (271.60/337.90)	306.80 (271.90/342.50)

^1^ Median (25th and 75th percentiles); * statistical significance was tested using Mann–Whitney U test; (*p* < 5 × 10^−2^).

**Table 3:** Bray–Curtis and Jaccard adjusted beta diversity between the neurological cases and healthy controls.

	Bray–Curtis			Jaccard		
	R^2^	*p*-Value	Significance	R^2^	*p*-Value	Significance
Medication regularly	4.04 × 10^−3^	9.99 × 10^−4^	***	6.74 × 10^−3^	9.99 × 10^−4^	***
Smoking habits	3.26 × 10^−3^	9.99 × 10^−4^	***	3.77 × 10^−3^	9.99 × 10^−4^	***
Alcohol	1.78 × 10^−3^	1.47 × 10^−1^	-	1.96 × 10^−3^	1.28 × 10^−1^	-
Dietary fiber	3.44 × 10^−3^	9.99 × 10^−4^	***	3.35 × 10^−3^	2.99 × 10^−3^	**
Docosahexaenoic acid	2.12 × 10^−3^	3.10 × 10^−2^	*	2.20 × 10^−3^	8.19 × 10^−2^	-
Vitamin B12	2.57 × 10^−3^	9.99 × 10^−4^	***	2.77 × 10^−3^	1.19 × 10^−2^	*
Vitamin B3	2.06 × 10^−3^	3.99 × 10^−2^	*	2.12 × 10^−3^	1.08 × 10^−1^	-
Vitamin D	1.69 × 10^−3^	2.01 × 10^−1^	-	1.79 × 10^−3^	2.00 × 10^−1^	-
BMI	4.13 × 10^−3^	9.99 × 10^−4^	***	4.61 × 10^−3^	9.99 × 10^−4^	***
Age	4.10 × 10^−3^	9.99 × 10^−4^	***	4.99 × 10^−3^	9.99 × 10^−4^	***
Sex	2.63 × 10^−3^	2.99 × 10^−3^	**	2.42 × 10^−3^	3.40 × 10^−2^	*
Group membership	3.31 × 10^−3^	9.99 × 10^−4^	***	1.03 × 10^−2^	9.99 × 10^−4^	***

Statistical significance was tested using permutation analysis of variance (PerMANOVA); (* *p* < 5 × 10^−2^, ** *p* < 1 × 10^−2^ and *** *p* < 1 × 10^−3^).

**Table 4:** Bray–Curtis and Jaccard adjusted beta diversity between the neurological subgroups and healthy controls.

	Bray–Curtis			Jaccard		
	R^2^	*p*-Value	Significance	R^2^	*p*-Value	Significance
Medication regularly	3.95 × 10^−3^	9.99 × 10^−4^	***	5.99 × 10^−3^	9.99 × 10^−4^	***
Smoking habits	3.26 × 10^−3^	9.99 × 10^−4^	***	3.69 × 10^−3^	9.99 × 10^−4^	***
Alcohol	1.76 × 10^−3^	1.64 × 10^−1^	-	1.82 × 10^−3^	1.99 × 10^−1^	-
Dietary fiber	3.65 × 10^−3^	9.99 × 10^−4^	***	3.67 × 10^−3^	1.99 × 10^−3^	**
Docosahexaenoic acid	2.10 × 10^−3^	2.99 × 10^−2^	*	2.29 × 10^−3^	5.39 × 10^−2^	
Vitamin B12	2.56 × 10^−3^	3.99 × 10^−3^	**	2.67 × 10^−3^	2.49 × 10^−2^	*
Vitamin B3	2.16 × 10^−3^	2.59 × 10^−2^	*	2.37 × 10^−3^	3.49 × 10^−2^	*
Vitamin D	1.69 × 10^−3^	2.16 × 10^−1^	-	1.74 × 10^−3^	2.32 × 10^−1^	-
BMI	4.29 × 10^−3^	9.99 × 10^−4^	***	4.79 × 10^−3^	9.99 × 10^−4^	***
Age	4.07 × 10^−3^	9.99 × 10^−4^	***	4.92 × 10^−3^	9.99 × 10^−4^	***
Sex	2.77 × 10^−3^	1.99 × 10^−3^	**	2.52 × 10^−3^	3.29 × 10^−2^	*
Group membership	8.09 × 10^−3^	9.99 × 10^−4^	***	1.71 × 10^−2^	9.99 × 10^−4^	***

Statistical significance was tested using permutation analysis of variance (PerMANOVA); (* *p* < 5 × 10^−2^, ** *p* < 1 × 10^−2^ and *** *p* < 1 × 10^−3^).

## Data Availability

Data sharing is not applicable to this article.

## Supplementary Materials

The following are available online at https://www.mdpi.com/article/10.3390/nu13113743/s1, Figure S1: Venn diagram of OTU occurrence in cases and controls (overlap of 144 OTUs, 5 OTUs (mainly *Proteobacteria*) and 42 OTUs (mainly *Firmicutes*) only observed in cases and controls). Figure S2: Linear discriminant analysis (LDA) effect size (LEfSe) plot of functional pathways identified in the gut microbiome of subgroups and controls. The threshold for the LDA score was 3 (*p* < 5 × 10^−2^). Figures and calculations were assessed by the “microeco” package (version 0.4.0) with the “Tax4Fun” package included in RStudio (version 0.3.1). SNA: structural neurological abnormalities. Table S1: Supplemental characterization of energy adjusted macro- and micronutrient intake between the groups.
