# Supplementary material for: BMI, Alcohol Consumption and Gut Microbiome Species Richness Are Related to Structural and Functional Neurological Abnormalities
Source: Nutrients. 2021 Oct 23;13(11):3743. doi: 10.3390/nu13113743 (PMC8618843; doi:10.3390/nu13113743)
Supplement: Supplementary file 1 [file nutrients-13-03743-s001.zip › Figure S1 Venn diagramm.pdf]

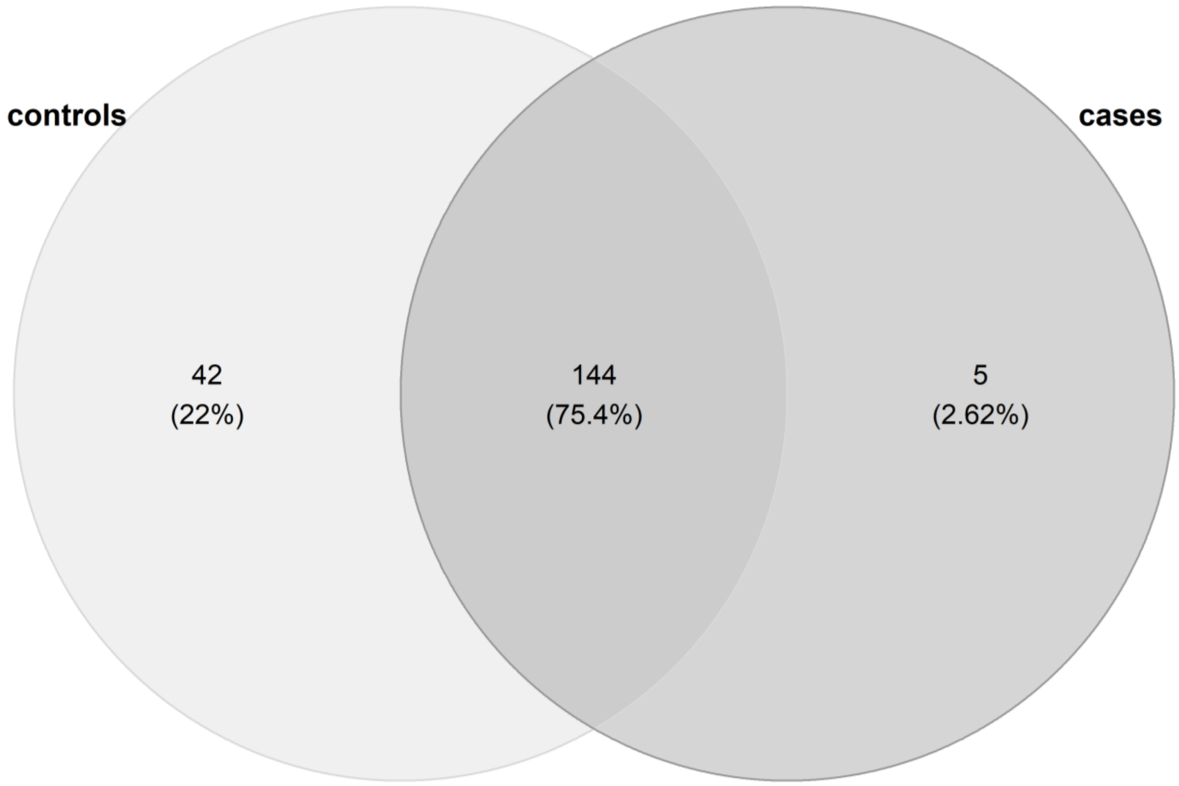

Figure S1 Venn diagram of OTU occurrence in cases and controls (overlap of 144 OTUs, 5 OTUs (mainly *Proteobacteria*) and 42 OTUs (mainly *Firmicutes*) only observed in cases and controls).
