# Supplementary material for: BMI, Alcohol Consumption and Gut Microbiome Species Richness Are Related to Structural and Functional Neurological Abnormalities
Source: Nutrients. 2021 Oct 23;13(11):3743. doi: 10.3390/nu13113743 (PMC8618843; doi:10.3390/nu13113743)
Supplement: Supplementary file 1 [file nutrients-13-03743-s001.zip › Figure S2 Linear discriminant analysis.pdf]

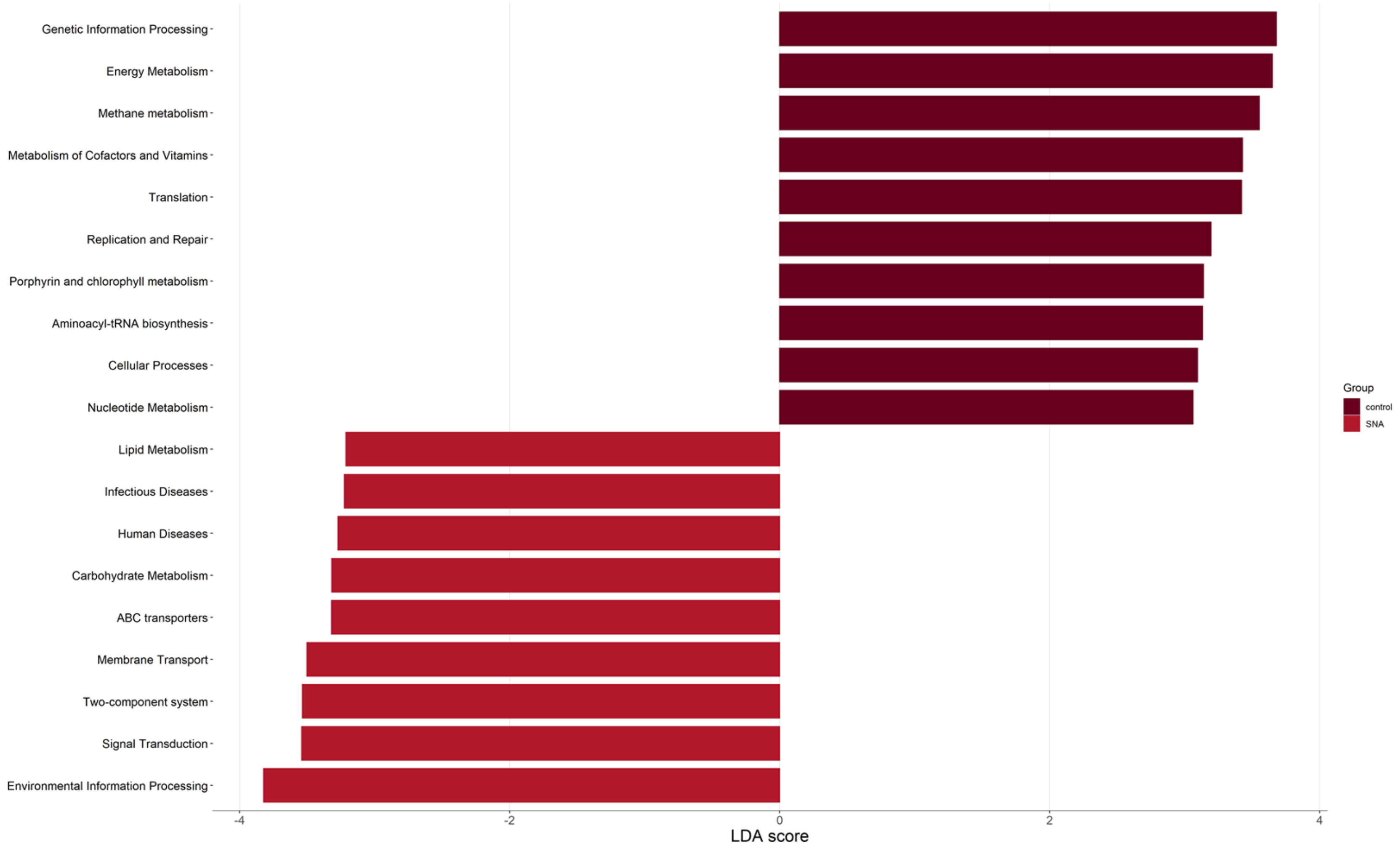

Figure S2 Linear discriminant analysis (LDA) effect size (LEfSe) plot of functional pathways identified in the gut microbiome of subgroups and controls. The threshold for the LDA score was 3 ($p < 5 \times 10^{-2}$). Figures and calculations were assessed by the "microeco" package (version 0.4.0) with the "Tax4Fun" package included in RStudio (version 0.3.1). SNA: structural neurological abnormalities.
