# Supplementary material for: BMI, Alcohol Consumption and Gut Microbiome Species Richness Are Related to Structural and Functional Neurological Abnormalities
Source: Nutrients. 2021 Oct 23;13(11):3743. doi: 10.3390/nu13113743 (PMC8618843; doi:10.3390/nu13113743)
Supplement: Supplementary file 1 [file nutrients-13-03743-s001.zip › Table S1 Supplement 1.pdf]

Table S1 Supplemental characterization of energy adjusted macro- and micronutrient intake between the groups.

	Cases (n=223)	Controls (n=568)	All subjects (n=791)
**Protein**			
**Isoleucine, g/d**	3.65 (3.26/3.95)[1]	3.62 (3.26/3.96)	3.60 (3.26/3.96)
**Leucine, g/d**	5.88 (5.27/6.35)	5.78 (5.26/6.38)	5.80 (5.26/6.38)
**Lysine, g/d**	5.07 (4.46/5.72)	5.02 (4.40/5.73)	5.03 (4.42/5.73)
**Threonine, g/d**	3.04 (2.75/3.32)	3.04 (2.74/3.38)	3.04 (2.74/3.37)
**Valine, g/d**	4.20 (3.80/4.51)	4.14 (3.78/4.57)	4.16 (3.78/4.56)
**Histidine, g/d**	2.01 (1.80/2.24)	1.99 (1.79/2.25)	2.00 (1.79/2.24)
**Alanine, g/d**	3.65 (3.17/4.11)	3.60 (3.21/4.03)	3.62 (3.20/4.05)
**Fatty Acids**			
**Butanoic acid/butyric acid, g/d**	1.29 (1.08/1.54)	1.34 (1.09/1.63)	1.32 (1.09/1.59)
**Hexanoic acid/caproic acid, g/d**	0.76 (0.62/0.92)	0.79 (0.63/0.97)	0.76 (0.62/0.92)
**Octanoic acid/caprylic acid, g/d**	0.72 (0.62/0.80)	0.73 (0.62/0.81)	0.72 (0.62/0.81)
**Decanoic acid/capric acid, g/d**	1.12 (0.96/1.29)	1.16 (0.97/1.35)	1.11 (0.97/1.29)
**Dodecanoic acid/lauric acid, g/d**	2.33 (2.03/2.63)	2.33 (2.07/2.62)	2.33 (2.03/2.63)
**Tetradecanoic acid/myristic acid, g/d**	4.69 (4.02/5.35)	4.79 (4.13/5.49)	4.69 (4.02/5.35)
**Pentadecanoic acid, g/d**	0.41 (0.34/0.50)	0.43 (0.34/0.52)	0.41 (0.35/0.49)
**Hexadecanoic acid/palmitic acid, g/d**	19.02 (17.29/21.14)	19.26 (17.55/21.03)	19.21 (17.42/21.06)
**Heptadecanoic acid, g/d**	0.35 (0.29/0.41)	0.36 (0.29/0.43)	0.36 (0.29/0.42)
**Octadecanoic acid/stearic acid, g/d**	8.10 (7.27/9.08)	8.17 (7.36/8.92)	8.16 (7.33/8.94)
**Eicosanoic acid/arachinic acid, g/d**	0.38 (0.34/0.41)	0.37 (0.33/0.41)	0.38 (0.34/0.41)
**Decosanoic acid, g/d**	0.11 (0.08/0.14)	0.09 (0.08/0.13)	0.10 (0.08/0.13)
**Teracosanoic acid, g/d**	0.02 (0.02/0.03)	0.02 (0.02/0.03)	0.02 (0.02/0.03)
**Octadecenoic acid/oleic acid, (g/day)**	29.59 (27.09/32.67)	29.58 (26.71/32.47)	29.59 (26.78/32.50)
**Dietary fiber**			
**Poly-hexoses, g/d**	6.71 (5.94/7.58)	6.55 (5.82/7.42)	6.58 (5.86/7.48)
**Poly-pentoses, g/d**	5.14 (4.42/6.19)	5.20 (4.30/6.15)	5.18 (4.33/6.18)
**Poly-uronic acid, g/d**	3.65 (2.90/4.81)	3.48 (2.73/4.44)	3.52 (2.77/4.53)
**Cellulose, g/d**	4.68 (3.95/5.79)	4.67 (3.92/5.52)	4.67 (3.93/5.58)
**Lignin, g/d**	1.36 (1.15/1.65)	1.32 (1.11/1.60)	1.33 (1.11/1.61)

[1] Median (25th and 75th percentiles); *statistical significance was tested using Mann-Whitney U test; ($p < 5 \times 10^{-2}$).
